# The Prophylactic Effect of Probiotic *Enterococcus lactis* IW5 against Different Human Cancer Cells

**DOI:** 10.3389/fmicb.2015.01317

**Published:** 2015-11-26

**Authors:** Yousef Nami, Babak Haghshenas, Minoo Haghshenas, Norhafizah Abdullah, Ahmad Yari Khosroushahi

**Affiliations:** ^1^Institute of Biosciences, Universiti Putra MalaysiaSelangor, Malaysia; ^2^School of Medicine, Shahid Beheshti University of Medical SciencesTehran, Iran; ^3^Chemical and Environmental Engineering Department, Faculty of Engineering, Universiti Putra MalaysiaSelangor, Malaysia; ^4^Drug Applied Research Center, Tabriz University of Medical SciencesTabriz, Iran; ^5^Department of Pharmacognosy, Faculty of Pharmacy, Tabriz University of Medical SciencesTabriz, Iran

**Keywords:** anticancer, enzyme activity, antibiotic susceptibility, apoptosis, cytotoxicity

## Abstract

*Enterococcus lactis* IW5 was obtained from human gut and the potential probiotic characteristics of this organism were then evaluated. Results showed that this strain was highly resistant to low pH and high bile salt and adhered strongly to Caco-2 human epithelial colorectal cell lines. The supernatant of *E. lactis* IW5 strongly inhibited the growth of several pathogenic bacteria and decreased the viability of different cancer cells, such as HeLa, MCF-7, AGS, HT-29, and Caco-2. Conversely, *E. lactis* IW5 did not inhibit the viability of normal FHs-74 cells. This strain did not generate toxic enzymes, including β-glucosidase, β-glucuronidase, and *N*-acetyl-β-glucosaminidase and was highly susceptible to ampicillin, gentamycin, penicillin, vancomycin, clindamycin, sulfamethoxazol, and chloramphenicol but resistant to erythromycin and tetracyclin. This study provided evidence for the effect of *E. lactis* IW5 on cancer cells. Therefore, *E. lactis* IW5, as a bioactive therapeutics, should be subjected to other relevant tests to verify the therapeutic suitability of this strain for clinical applications.

## Introduction

Probiotics are non-pathogenic live microorganisms that provide health benefits when these organisms are consumed in sufficient amounts (FAO/WHO, 2001; Mehra et al., 2012; Howarth and Wang, 2013; Haghshenas et al., 2014a; Nami et al., 2014a). Probiotics have been utilized to prevent bacterial infections (Forsyth et al., 2009) and treat cancer (Baldwin et al., 2010; Haghshenas et al., 2014b, 2015a,b; Nami et al., 2014b,c,d). These organisms can also create an acidic environment in the colon by producing short-chain fatty acids. Furthermore, probiotic bacteria can inhibit the occurrence of cancer by (i) lowering pH, (ii) reducing the level of pro-carcinogenic enzymes (Donaldson, 2004), (iii) enhancing cell proliferation by inhibiting normal cell apoptosis and by promoting cell differentiation and cytoprotective activities (Lin et al., 2008), and (iv) suppressing inflammation-induced cell apoptosis (Prisciandaro et al., 2011) caused by lactic acid bacteria (LAB), including *Lactobacillus, Enterococcus, Streptococcus*, and *Bifidobacterium*. Among these LABs, the genus *Enterococcus* has gained considerable interest in environmental, food, and clinical research (Sharma et al., 2012).

*Enterococcus* is ubiquitous in nature and considered as the most controversial LAB genus because of unclear functions (Galvez et al., 2009). Enterococci have been utilized as adjutants to treat human and animal diseases. Enterococci have also been used in the food industry as probiotics (Franz et al., 2003) or as starter cultures because these microorganisms produce useful bacteriocins (Fisher and Phillips, 2009). Although *Enterococcus* comprises many species, only a few species are recognized as probiotics, such as *E. faecalis, E. faecium*, and *E. lactis*. Probiotics should exhibit important characteristics, such as tolerant to gastrointestinal conditions (acid and bile) and non-pathogenic; probiotics should also display competitive exclusion of pathogens (Collins et al., 1998; Ouwehand et al., 2002). Thus, the selection criteria of probiotic bacteria for clinical applications should be carefully evaluated. This study aimed to determine the probiotic properties (bile tolerance, antimicrobial activity, and antibiotic susceptibility) and antitumor activities of *E. lactis* isolated from the human gut.

## Materials and Methods

### Bacterial Strain and Culture Condition

*Enterococcus lactis* IW5 was isolated from human fecal samples using streak plate method previously described by Shin et al. (2015) and this strain was maintained at -70°C in de Man Rogosa broth (MRS, Merck, Germany) containing 25% (v/v) glycerol. *E*. *lactis* IITRHR1 isolated from cheese was used as a control strain. Working cultures were anaerobically incubated at 37°C for 24 h in an anaerobic jar (Mitsubishi Inc. USA) that contains anaerobic gas generation kits (AnaeroPack).

### Tolerance to Artificial Gastric Juice and Artificial Bile Acid

Tolerance to artificial gastric juice and bile acid were determined according to previously described method with slight modification (Lee et al., 2014). *E. lactis* was suspended in MRS containing 0.1% pepsin (Sigma, St. Louis, MO, USA) and adjusted to a pH of 2.0 with 0.1 M HCl, and then incubated for 3 h at 37°C. Artificial bile acid tolerance was measured by cultivating cells treated with artificial gastric juice. The cells were incubated at 37°C for 24 h in artificial bile acid consisting of MRS containing 0.3% oxgall (Becton Dickinson, Sparks, MD, USA). The numbers of viable cells were measured by incubating aliquots for 24 h on MRS agar plates at 37°C. The survival rate was calculated using the formulation:

Survival rate (%) = (Log CFU after reaction/Log CFU at 0 h) × 100

### Antimicrobial Susceptibility Assay

Thirteen pathogenic organisms from the Persian Type Culture Collection (**Table 1**) were selected to detect antagonistic substances. Well diffusion was performed to detect inhibitory substances produced in the supernatant fluid of the isolate. For this purpose, an overnight culture of the indicator strains was used to inoculate appropriate agar growth media (Dimitonova et al., 2007) at 37°C. Wells with a diameter of 5 mm were cut into agar plates; afterward, 50 μL of filtered cell-free supernatant obtained from the third subculture of the microorganisms grown in MRS broth (cell density 10^8^ cfu/mL) was added to each well. The supernatant was obtained by growing inhibitory producer strains overnight in MRS broth at 37°C. The cells were removed through centrifugation; the supernatant was placed in the wells and allowed to diffuse in agar for 2 h at room temperature. The plates were incubated at optimum growth temperature of the indicator strains and examined after 24 h to determine inhibition zone areola diameter (Nowroozi et al., 2004; Maldonado et al., 2012).

**Table 1:** The inhibitory effect of *Enterococcus lactis* IW5 against pathogenic bacteria.

Test organisms	Growth conditions	Origin	Susceptibility
*Salmonella typhimurium* *Escherichia coli* O26 *E. coli* O157 *Staphylococcus aureus* *Bacillus cereus* *Listeria monocytogenes* *Klebsiella pneumoniae* *Shigella flexneri* *Pseudomonas aeroginosa* *Candida albicans* *Serratia marcesens* *Streptococcus mutans* *Staphylococcus saprophyticus*	MPA, 37°C LB, 37°C LB, 37°C Blood agar, 37°C MPA, 37°C BHI, 37°C MPA, 37°C MHA, 37°C MPA, 37°C MHA, 28°C MHA, 37°C MHA, 37°C Blood agar, 37°C	ATCC 14028 Native strain PTCC 1276 ATCC 25923 PTCC 1539 (ATCC 11778) PTCC 1163 PTCC 1053 (ATCC 10031) PTCC 1234 (NCTC 8516) PTCC 1181 PTCC 5027 (ATCC 10231) PTCC 1187 (Native strain) PTCC 1683 (ATCC 35668) PTCC 1440 (CIP 76.125)	R S R S S ES S S R R R SS R

*R: 0 mm; SR: 0–4 mm; SS: 4–8 mm; S: 8–12 mm; ES: >12 mm.*

*CIP, Collection of Bacteries de l’Institute Pasteur, Paris, France; ATCC, American Type Culture Collection, Virginia, USA; NCTC, National Collection of Type Cultures, London, UK; PTCC, Persian Type Culture Collection, Tehran, Iran.*

*MPA, mycophonolic acid; LB, Lysogeny broth; BHI, Brain-heart infusion medium; MHA, Mueller Hinton Agar.*

### Enzyme Activity

Enzyme activity was evaluated using an API ZYM kit (BioMerieux, Paris, France). *E. lactis* IW5 was suspended in sterile saline (0.85% NaCl) at 10^5^ CFU/mL and added to each cupule. After inoculation was performed, the cultures were incubated at 37°C for 4 h. One drop of ZYM B reagent was added and a drop of surface-active agent (ZYM reagent) was added to each cupule. ZYM A was introduced to facilitate ZYM B solubilization in the medium. The resulting color was observed for at least 5 min. Values ranging from 0 to 5 were assigned on the basis of color strength to determine the approximate amount (in nmol) of hydrolyzed substrate.

### Cell Cultures

Five human cancer lines, namely, Caco-2 (human colorectal carcinoma cell), AGS (human gastric carcinoma cell), MCF-7 (human breast carcinoma cell), HeLa (human cervical carcinoma cell), and HT-29 (human colon carcinoma cell), and one normal cell line, namely, FHs-74 (human intestinal epithelial cells) – obtained from cell resource center of Pasteur institute of Iran (Tehran, Iran) – were used to investigate the anticancer effects of *E. lactis* IW5. The cells were grown in RPMI-1640 medium supplemented with 10% heat-inactivated fetal bovine serum and a 1% penicillin–streptomycin mixture. The cultures were maintained at 37 °C in an atmosphere of 95% O_2_ and 5% CO_2_ with relative humidity (Merghoub et al., 2009).

### Cell-free Culture Supernatant Preparation

The liquid culture of *E. lactis* at the end of the exponential growth phase was centrifuged at 4000 × *g* for 10 min to obtain cell precipitates. The supernatant was collected; pH was adjusted to 7.2 with 1 N NaOH and subjected to lyophilization. Endogenous proteases were inactivated by heat at 100 C for 3–5 min. The desired concentrations of lyophilized culture supernatant (10–50 μg/mL) were prepared in RPMI media by diluting from stock solution (10 mg lyophilized supernatant/mL RPMI media) and sterilized by filtering the supernatant through a 0.22 μm bacterial filter (Millipore); the prepared supernatant was then used to treat cancer cells.

### Adhesion to Caco-2 Cells

*Enterococcus lactis* IW5 was assessed for its adhesion ability to the human colon carcinoma cell line, Caco-2. The cells were seeded in RPMI-1640 medium supplemented with 10% heat-inactivated fetal bovine serum and 1% penicillin/streptomycin mixture. Cells were seeded on 24-well tissue culture plates and incubated at 37°C in 5% CO_2_ in a relatively humid atmosphere until a confluent monolayer was achieved. Adherence assay was carried out by adding 1 mL of the bacterial strain, suspended in RPMI-1640 medium, at a concentration of about 1 × 10^7^ CFU/well and was incubated for 3 h at 37°C in an atmosphere of 5% (v/v) CO_2_. Before the adhesion assay, the media in the wells containing a Caco-2 cell monolayer were removed and replaced once with fresh antibiotic-free RPMI.

To remove non-attached bacterial cells, the wells were washed three times with a sterile, pre-warmed PBS solution. To detach the cells from the wells, 1 mL of trypsin/EDTA solution (0.5% porcine trypsin and 0.2% EDTA in PBS; Sigma) was added to each well and the mixture was gently stirred for 5 min. To measure the viable Caco-2 cell count, the cells were counted by the pure plate method onto MRS agar medium and incubated at 37°C under anaerobic conditions. Bacterial adhesion was expressed as the total number of bacteria attached to viable Caco-2 cells.

### Cytotoxicity against Different Cancer Cells

The cytotoxicity of the isolated *E. lactis* on tumor/normal cells was evaluated through a microculture tetrazolium [MTT, 3-(4, 5-dimethylthiazol-2-yl)-2,5-diphenyltetrazolium bromide] assay (Mosmann, 1983). In brief, HeLa, AGS, MCF-7, HT-29, Caco-2, and FHs 74 cells (1.2 × 10^4^ cells/well) were seeded in each well of a 96-well microplate with RPMI growth medium. Once 50% confluence was reached 24 h after the cells were seeded, the cells were treated with the filtered supernatant of the isolated strain at different time points (12, 24, and 48 h). After treatment was administered, the medium was replaced with 200 μl of fresh medium containing 50 μl of MTT solution (2 mg/mL in PBS) and incubated for another 4 h at 37 C. After incubation was completed, the MTT mixture was carefully removed, and 200 μl of dimethyl sulfoxide and 25 μl of Sorenson’s glycine buffer (0.1 M glycine and 0.1 M NaCl at pH 10.5) were added to each well and incubated for 30 min. The absorbance of each well was determined after 30 s of shaking by using a microplate reader (Biotek, ELx 800, USA) at 570 nm. The cells treated with MRS (bacterial culture medium) and Taxol (anticancer drug as a reference) served as negative and positive controls, respectively.

### Apoptotic Cells Detection

#### 4′,6-diamidino-2-phenylindole (DAPI) staining

All of the cultured cells (treated/untreated groups) were evaluated through 4′,6-diamidino-2-phenylindole (DAPI) staining to detect apoptotic cells. For this purpose, sterile cover slips were placed in each of the six wells of the culture plate. Cancer cells (120 × 10^4^ cells/well) were added to each well and maintained under the desired standard culture condition. At 24 h after the cells were seeded, all of the cultured cells were subjected to *E. lactis* secretion, MRS medium, and Taxol (IC_50_ concentration) treatments. The treated and untreated control groups were incubated for another 24 h and prepared for apoptosis assay. Afterward, 4% paraformaldehyde was added to each well to stain cells with DAPI dye. The cells were fixed and permeabilized with 0.1% Triton-X100 for 5 min. The permeabilized cells were stained with 50 μl of DAPI dye (1:2000 dilutions) and incubated for 3 min at room temperature. The processed cells with cover slips were washed thrice with PBS (pH 7.2) and utilized to assess apoptosis by using a fluorescent microscope (BX64, Olympus, Japan) equipped with a U-MWU2 fluorescence filter (excitation filter BP 330-385, dichromatic mirror DM 400, and emission filter LP 420; Paolillo et al., 2009).

#### Flow cytometry

The fraction of apoptotic cells was quantitatively measured via flow cytometry using the Annexin V-FITC apoptosis detection kit (eBioscience, San Diego, CA, USA). HeLa cell line (1.2 × 10^5^ cells/well) was seeded into a six-well culture plate and the treatment of cells were similar to DAPI staining. After treatment time point (24 h), the treated/untreated control cells were detached by trypsin, the supernatant was discarded after centrifugation at 900 rpm for 10 min at 28 C, and the cell pellet was resuspended in 500 μl of 1× binding buffer and transferred into a new 5 ml tube. The tubes were centrifuged again and the supernatants were replaced with100 μl binding buffer (1×). Afterward, the tubes were added with 5 μl of FITC-conjugated Annexin V then were incubated for 15 min at room temperature under dark conditions. The incubated cells were centrifuged and the cell plates were resuspended in 500 μl of binding buffer (1×). Finally, 5 μl of propidium iodide solution was added to the cells, and quadrant settings were fixed with untreated, single-stained controls, and copied to dot plots of the treated cells. Quadrant statistic calculations were performed using CELLQuest Pro software (BD Biosciences, San Jose, CA, USA). The experiment was repeated two times with triplicate samples for each experiment. Analyses were accomplished using 10000 cells at a rate of 450 cells/s.

### Quantitative Real Time PCR

For RNA analysis, HeLa cells were lysed using TRI Reagent^®^(Sigma Chemical Co., Poole, UK) according to manufacture guidelines. 24 h post-treatment or untreated control monolayer cells were lysed by adding desired amount of TRI Reagent^®^ (2 mL per 25 cm2 T-flask) accordingly were homogenized and transferred to RNAse/DNAse-free microtubes. Chloroform (0.2 mL per each mL of TRI Reagent^TM^ used for lysing) was added to each sample, and the mixture was vortexed. After maintaining at room temperature for 5 min, the samples were centrifuged at 12000 × *g*, 4°C and 10 min and the colorless supper aqueous phase was carefully separated and mixed with ice-cold isopropanol (0.5 mL per each mL of TRI Reagent^®^ used initially). The mixture was centrifuged at12000 × *g*, 4°C for 10 min, yielding total RNA pellet that was washed with 75% ethanol (×3). The air dried samples were dissolved in DEPC treated water and tested qualitatively and quantitatively prior to its use for RT-PCR experiments.

The isolated RNA was reverse transcribed to cDNA using Moloney- murine leukemia virus (MMLV) reverse transcriptase (Bethesda Research Laboratories, Gaithersburg, MD, USA). For RT reaction, 1 μL RNA (1 μg/μL) was mixed with master mix [DEPC treated water 13 μL, dNTP’s (10 μM) 2 μL, MMLV buffer with DTT 2 μL, random hexamer primer (pdN6; 400 ng/μL) 0.5 μL], and denatured at 95°C for 5 min. The sample was then cooled down to 4°C for 5 min using ice-bath. Then 1 μL MMLV (200 U/μL) and 0.5 μL RNase in (40 U/μL) were added to the sample and the mixture was incubated using following thermocycling program: 10 min at 25°C, 42 min at 42°C, and 5 min at 95°C. The prepared cDNA templates were used for real time PCR experiments.

Primers were designed from published Gene Bank sequences using Beacon Designer 5.01 (Premier Biosoft International, http://www.premierbiosoft.com) and listed in **Table 3**. All amplification reactions were performed in a total volume of 25 μL using iQ5 Optical System (Bio-Rad Laboratories Inc., Hercules, CA, USA). Each well contained: 1 μL cDNA, 1 μL primer (100 nM each primer), 12.5 μL 2× Power SYBR Green PCR Master Mix (Applied Biosystems, Foster City, CA, USA), and 10.5 μL RNAse/DNAse free water. Thermal cycling conditions were as follow: 1 cycle at 94°C for 10 min, 40 cycles at 95°C for 15 s, 56–62°C (annealing temperature) for 30 s, and 72°C for 25 s. Interpretation of the result was performed using the Pfaﬄe method and the threshold cycle (*C*_t_) values were normalized to the expression rate of GAPDH as a housekeeping gene. All reactions were performed in triplicate and negative controls were included in each experiment.

### Statistical Analysis

Data were analyzed by one-way ANOVA. Significant differences of means (*p* < 0.05) were compared through Duncan’s test by using SPSS 19.0. Graphs were prepared using Microsoft Office Excel (Rahmati, 2011).

## Results and Discussion

### Isolation and Identification

The bacterial strain was isolated from the human gut. The strain was initially identified by phenotypic methods; the Gram reaction of the isolates was determined by observation under a light microscope after Gram staining by using a Gram staining kit. LAB were considered Gram positive when they appeared blue–purple upon Gram staining. The isolates did not produce gas bubbles when hydrogen peroxide solution (3%) drops (Sigma–Aldrich, USA) were added to bacterial cells to determine catalase positive/negative strains; hence, the result confirmed that this strain is a Gram-positive and catalase-negative bacterium. A total of 45 Gram-positive and catalase-negative strains were obtained. Based on 16S rRNA identification results, the 45 isolated bacteria were classified into three major groups of LAB: enterococci, lactobacilli, and lactococci. After sequencing was performed, the strains belonging to *Enterococcus* genus were categorized into nine different species: one *E. lactis*, two *E. pseudoavium*, four *E. hirae*, two *E. gilvus*, four *E. avium*, three *E. durans*, eight *E. faecalis*, five *E. malodoratus*, and seven *E. faecium*. Moreover, lactobacilli were classified into three diverse species: one *L. casei*, three *L. acidophilus*, and one *L. plantarum*. Lactococci were classified into one species: three *Lactococcus lactis*, with two subspecies, namely, *L. lactis* ssp. *lactis* and *L. lactis* ssp. *cremoris*.

Probiotics have been extensively investigated because these organisms provide health benefits when such probiotics are consumed in sufficient amounts. In this study, LAB species with probiotic and antitumor activities were isolated; the strains that could grow in 5% CO_2_ atmosphere. An *E. lactis* strain (Accession number: HF562969.1) resistant to pH 2.0 and 0.3% bile salt was isolated from the human gut and then identified.

### Acid and Bile Tolerance

The survival of *E. lactis* IW5 and *E. lactis* IITRHR1 in artificial gastric juice (pH 2.0,0.1% pepsin, for 3 h) and artificial bile salt (0.3% oxgall, for 24 h)was evaluated (**Table 1**). The cells of *E. lactis* IW5 and *E. lactis* IITRHR1 were strongly maintained, with 94.60 and 92.27% survival rate in artificial gastric juice, respectively. In artificial bile salt, the cells of *E. lactis* IW5 and *E. lactis* IITRHR1demonstrated 95.46 and 94.14% survival rate, respectively. Our findings are similar to those of previous studies, which revealed that the survival rates of *Enterococcus* bacteria treated with acid and bile range from 63 to 100% (Haghshenas et al., 2014b; Nami et al., 2014d). Similarly, it has been revealed that *Enterococcus* bacteria were very stable in acidic conditions (pH 2 for 3 h) and high bile salt (0.3% oxgall for 4 h; Bhardwaj et al., 2010).

### Antimicrobial Susceptibility Assay

The antimicrobial susceptibility spectrum of *E. lactis* IW5 is shown in **Table 2**. This strain inhibited the growth of pathogenic bacteria, including *Escherichia coli* O26, *Staphylococcus aureus, Bacillus cereus, Klebsiella pneumoniae, Shigella flexneri*, and *Streptococcus mutans*. Moreover, *E. lactis* exhibited strong activity against *Listeria monocytogenes*. No significant activity was observed against *Serratia marcesens, Pseudomonas aeruginosa, Candida albicans, Staphylococcus saprophyticus, Escherichia coli* O157, and *Salmonella typhimurium*.

**Table 2:** Tolerance of *E. lactis* IW5 and *E. lactis* IITRHR1 against artificial gastric and bile conditions.

Treatment	Log CFU/mL
***E. lactis* IW5** Initial cell no. pH 2.0, 0.1% pepsin, 2 h 0.3% oxgall, 24 h	8.15 ± 0.26 7.71 ± 0.12 7.78 ± 0.36
***E. lactis* IITRHR1** Initial cell no. pH 2.0, 0.1% pepsin, 2 h 0.3% oxgall, 24 h	8.36 ± 0.18 7.71 ± 0.21 7.87 ± 0.19

*The results are represented as mean ±*SD*.*

**Table 3:** Real time PCR genes and their forward/reverse primers.

Primer	Forward and reverse primer	Sequence	Amplicon size	length
BAX	F	5′-CCCGAGAGGTCTTTTTCCGAG-3′	155	21
	R	5′-CCAGCCCATGATGGTTCTGAT-3′	155	21
BCL2	F	5′-GGTGGGGTCATGTGTGTGG-3′	130	19
	R	5′-CGGTTCAGGTACTCAGTCATCC-3′	130	22
CASPAS 9	F	5′-CTCAGACCAGAGATTCGCAAAC-3′	116	22
	R	5′-GCATTTCCCCTCAAACTCTCAA-3′	116	22
CASPAS 8	F	5′-GACAGAGCTTCTTCGAGACAC-3′	116	21
	R	5′-GCTCGGGCATACAGGCAAAT-3′	116	20
ErbB2	F	5′-TGTGACTGCCTGTCCCTACAA-3′	152	21
	R	5′-CCAGACCATAGCACACTCGG-3′	152	20
ErbB3	F	5′-GACCCAGGTCTACGATGGGAA-3′	99	21
	R	5′-GTGAGCTGAGTCAAGCGGAG-3′	99	20
BCL-XL	F	5′-GAGCTGGTGGTTGACTTTCTC-3′	101	21
	R	5′-TCCATCTCCGATTCAGTCCCT-3′	101	21

The 50% inhibitory concentration (IC_50_) of isolated strain metabolites was determined as an index of antagonistic activity from the antimicrobial time and dose-dependent curves. After 24 h of incubation, IC_50_ values were only observed in *E. coli* O26, *S. aureus, B. cereus, K. pneumoniae, S. flexneri, S. mutans*, and *L. monocytogenes* cells treated with *E. lactis* secretions. The IC_50_ for *E. lactis* secretions on *E. coli* O26, *S. aureus*, and *B. cereus* cells was 47, 28 and 32 μg/mL, respectively. The IC_50_ values of *E. lactis* secretions on *K. pneumoniae, S. flexneri*, and *S. mutans* cells was 31, 26 and 22 μg/mL, respectively. The IC_50_ value of *E. lactis* secretions on *L. monocytogenes* cells showed the lowest value (13 μg/mL). Our results showed that the *E. lactis* IW5 strain obtained from the human gut exhibited good probiotic properties, such as low pH and bile salt resistance. This strain was capable to inhibit several pathogenic bacteria.

### Enzyme Activity

Certain enzymes are characteristically produced by probiotics to provide protection from toxic substances. β-glucosidase, *N*-acetyl-β-glucosaminidas, and β-glucuronidase have been associated with certain health disorders (Chen et al., 2014). β-glucuronidase increases the risk of carcinogenesis by secreting toxins and mutagens (Delgado et al., 2007; Dabek et al., 2008). These toxic enzymes could be produced by microorganisms. Our data demonstrated that *E. lactis* did not produce toxic enzymes, including β-glucosidase, *N*-acetyl-β-glucosaminidase, and β-glucuronidase. Conversely, *E. lactis* produced various enzymes, including esterase (20 nmol), acid and alkaline phosphatase (5 nmol), and esterase lipase (≥25 nmol).

### Adhesion Ability to Colon Endothelial Cells

Several investigations have implicated a number of factors in the attachment of probiotic bacterial cells to epithelial cells. Such factors include: passive entrapment of the bacterial cells by fimbrial cell matrix material (Sarem et al., 1996), bacterial cell surface-associated lipoteichoic acid (Granato et al., 1999), proteinaceous extracellular adhesins (Conway and Kjelleberg, 1989), and bacterial cell surface-associated proteinaceous factors (Adlerberth et al., 1996). Adhesion of *E. lactis* IW5 and *E. lactis* IITRHR1 was confirmed by using the plating technique. When *E. lactis* IW5 was plated at a concentration of 8.35 ± 0.06 log CFU/well, we found that 8.16 ± 0.04 log CFU/well of the bacteria adhered to the Caco-2 cells. Conversely, when *E. lactis* IITRHR1 was plated at a concentration of 8.13 ± 0.05 log CFU/well, it was found that only 6.45 ± 0.03 log CFU/well of the bacteria adhered to the Caco-2 cells. It has been reported previously that *E. lactis* IITRHR1 can strongly adhere to intestinal epithelial cells, which promote its survival and show a broad range of antimicrobial activity (Sharma et al., 2011). Similar to our findings, these data demonstrated that the bacterial concentration was reduced by 1.68 log CFU/well, following removal of the non-adhered cells.

### Toxicity Assay

**FIGURE 1:**
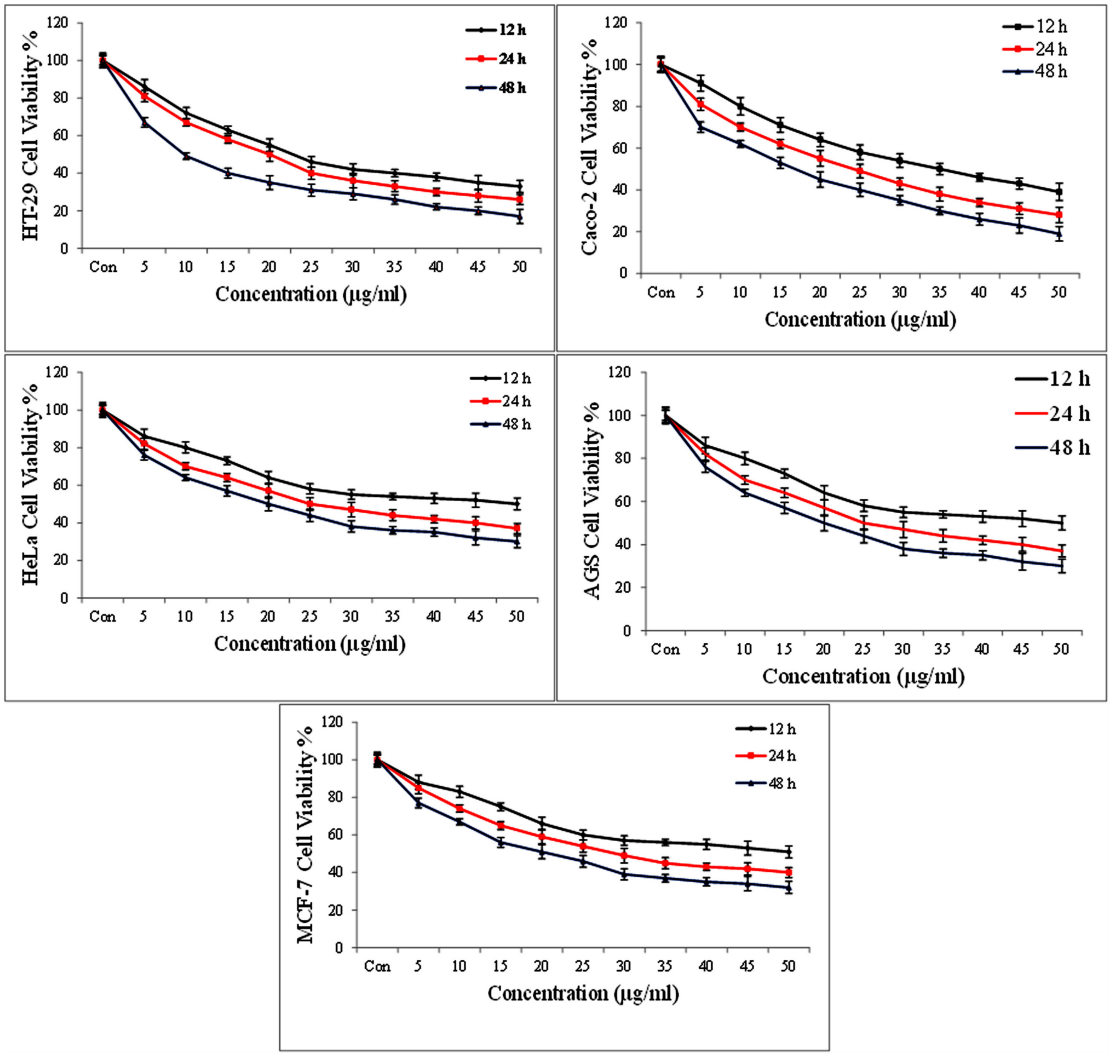
**The cytotoxic effects of isolated *Entreococcus lactis* IW5 secretion on different cancer cell lines at three time points 12, 24, and 48 h.** Error bars represent the standard deviation of the each mean.

**FIGURE 2:**
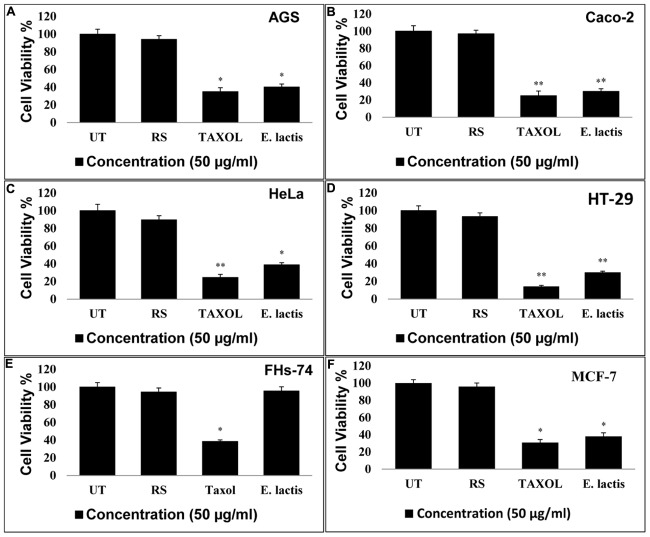
**Effect of *E. lactis* secretions on the proliferation of cancerous MCF-7, Caco-2, HT-29, HeLa, AGS, and FHs-74 normal cell lines.** *E. lactis* secretions final concentration: 50 μg/mL, Taxol concentration: IC_50_ for each cell line. Incubation time is 24 h. Data are expressed as mean and error bars represent standard deviation of means. UT: Untreated media were used as control. *E. lactis* IITRHR1 was used as Reference Strain (RS) for comparison. Asterisks denote statistically significant differences (^∗^*p < 0.05*; ^∗∗^*p < 0.01*).

Microculture tetrazolium assay was performed to determine the cytotoxicity effects of the metabolites secreted by *E. lactis* IW5 on various cancer cell lines, particularly HeLa, Caco-2, AGS, and HT-29. The cytotoxicity potential of the metabolites produced by *E. lactis* IW5 on various cancer cells was determined (**Figures 1** and **2A–D,F**). After 24 h of incubation, the metabolites inhibited all cancer cell lines. Approximately 38, 36, 28, 40, and 30% of MCF-7, HeLa, HT-29, AGS, and Caco-2 cells, respectively, remained viable after these cells were incubated with the metabolites for 24 h. The antiproliferative effect of the metabolites on all of the evaluated cancer cells significantly differed from that of the un-treated and reference strain-treated groups. The effect of the metabolites on FHs 74 normal cells was also examined (**Figure 2E**). *E. lactis* IW5 secretions exhibited no toxic effect on normal cells; more than 95% of the cells grew well. These results indicated that *E. lactis* IW5 is a potential candidate for cancer treatment.

The anticancer activity of probiotic bacteria has been demonstrated by *in vivo* and *in vitro* systems (Ouwehand, 2007). Probiotic organisms inhibit mammalian cell proliferation in primary leukocyte cultures and cell lines. The induction of apoptotic cells by conjugated linoleic acid produced by various probiotic strains has been established in Caco-2 and HT-29 mammalian cancer cell lines. In this study, four human cancer cell lines, namely, Caco-2 (colorectal cancer), AGS (gastric cancer), HeLa (cervical cancer), and HT-29 (colon cancer), and one normal cell line, namely, FHs-74, were utilized. The results of this study demonstrated that the metabolites secreted by *E. lactis* IW5 strain significantly inhibited the growth of the four cancer cell lines. *E. lactis* IW5 secretions decreased the proliferation and viability of all cancer cell lines but did not adversely affect FHs-74 normal cells. Therefore, this strain was considered non-toxic. Different cancer cells were treated with 10^6^ CFU/well of *E. lactis* IW5; this treatment strongly inhibited the proliferation of cancer cells. In *E. lactis* IW5 treatment, the proliferation of MCF-7, HeLa, HT-29, AGS, and Caco-2 cells was particularly inhibited by 38, 36, 28, 40, and 30%, respectively. Thus, *E. lactis* IW5 can inhibit the proliferation of cancer cells; however, *E. lactis* IITRHR1could not inhibit the proliferation of cancer cells.

### Apoptosis Assay

**FIGURE 3:**
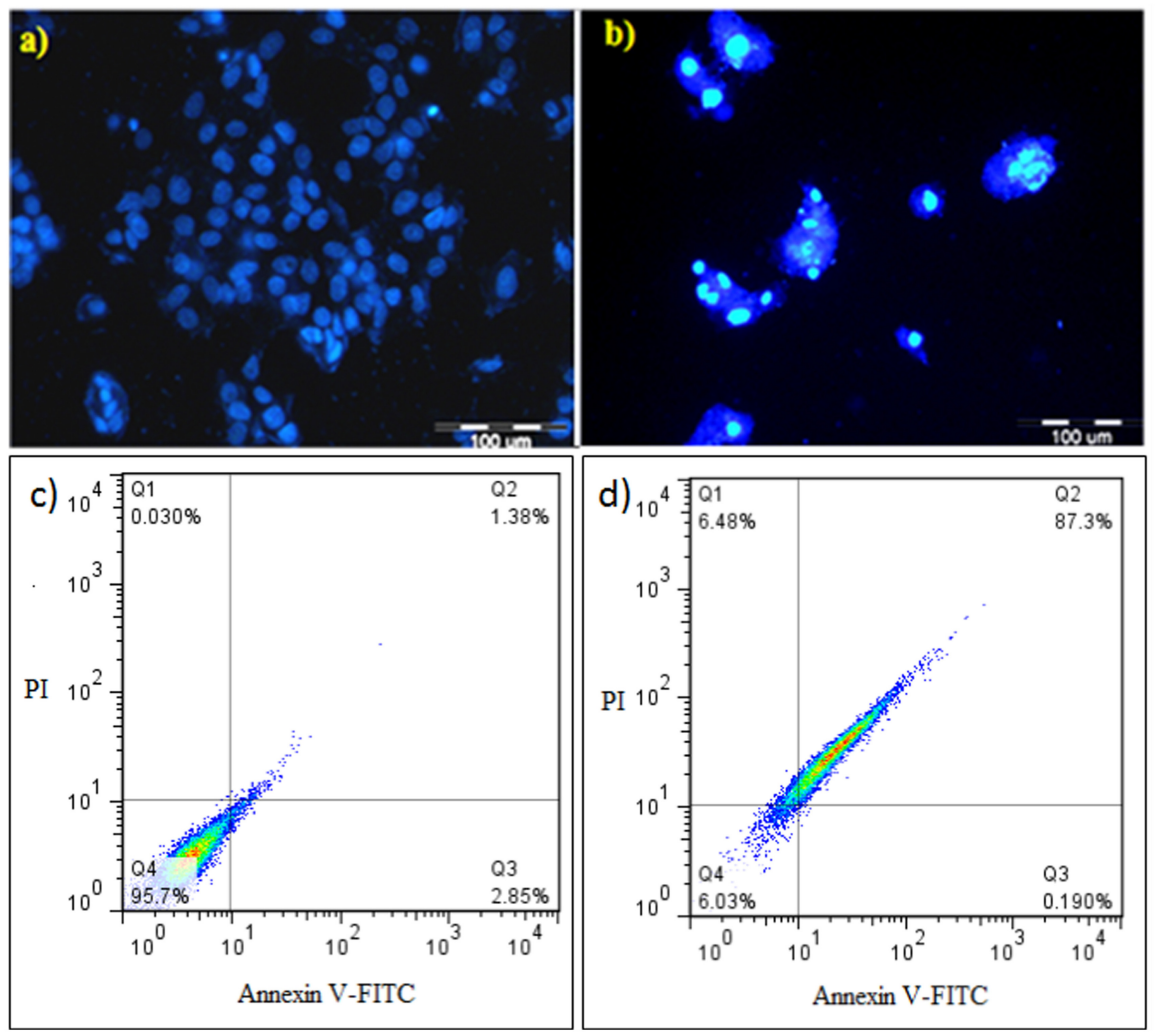
**4′,6-diamidino-2-phenylindole (DAPI) staining and flow cytometric analysis of treated/untreated HeLa cancer cells. (a,b)** Untreated and treated DAPI-stained cells; **(c,d)** flow cytometric analysis of untreated and treated cells with 50 μg/mL *E. lactis* secretion metabolites after 24 h incubation. Dots with Annexin V^-^/PI^+^ (Q1), Annexin V^+^/PI^+^ (Q2), Annexin V^+^/PI^-^ (Q3), and Annexin V^-^/PI^-^ (Q4) and feature represent necrotic, late apoptotic, early apoptotic, and viable intact cells, respectively.

HeLa cells were treated with 50 μg/mL of the filtered secretion after these cells were incubated for 24 h; the treated HeLa cells were stained with DAPI and analyzed through fluorescent microscopy to analyze the effect of *E. lactis* secretions on HeLa cell viability. The intact viable cells displayed completely healthy nuclei (**Figure 3a**); by contrast, the apoptotic cells were characterized by shrunk cells with condensed (early apoptosis) or fragmented (late apoptosis) nuclei. Other morphological and apoptotic changes, such as membrane blebbing and apoptotic body formation, were observed in the treated cells. This result suggested that apoptosis is the main cytotoxic mechanism of bacterial metabolites (**Figure 3b**). The newly identified *E. lactis* IW5 strain obtained from the human gut exhibited appropriate probiotic properties, such as high tolerance to low pH, resistance to high bile salt concentration, and anti-pathogenic activity against several pathogenic bacteria. Cytotoxic findings indicated that *E. lactis* IW5 secreted metabolites that possessed high anticancer activity against all of the examined cancer cell lines (AGS, Caco-2, HeLa, and HT-29). Therefore, the metabolites produced by *E. lactis* IW5 strain may be used as an alternative nutraceutical with promising therapeutic index because these metabolites are non-cytotoxic to normal mammalian cells.

Compared with the control cells that exhibited natural cell death (**Figure 3c**), the HeLa cells treated with 50 μL/mL of filtered *E. lactis* IW5 secretions demonstrated significant amounts (*p* ≤ 0.05) of annexin V^+^/PI^+^ (late apoptotic cells) after incubating for 24 h (**Figure 3d**). In the treated HeLa cells 87.3 and 6.48% were observed in late apoptosis and necrosis, respectively. Based on the flow cytometry findings, *E. lactis* IW5 secretions can inhibit the proliferation of cancer cells and the main mechanism of this prophylactic effect was related to apoptosis induction in cancer cells.

### Quantitative Real Time PCR

**FIGURE 4:**
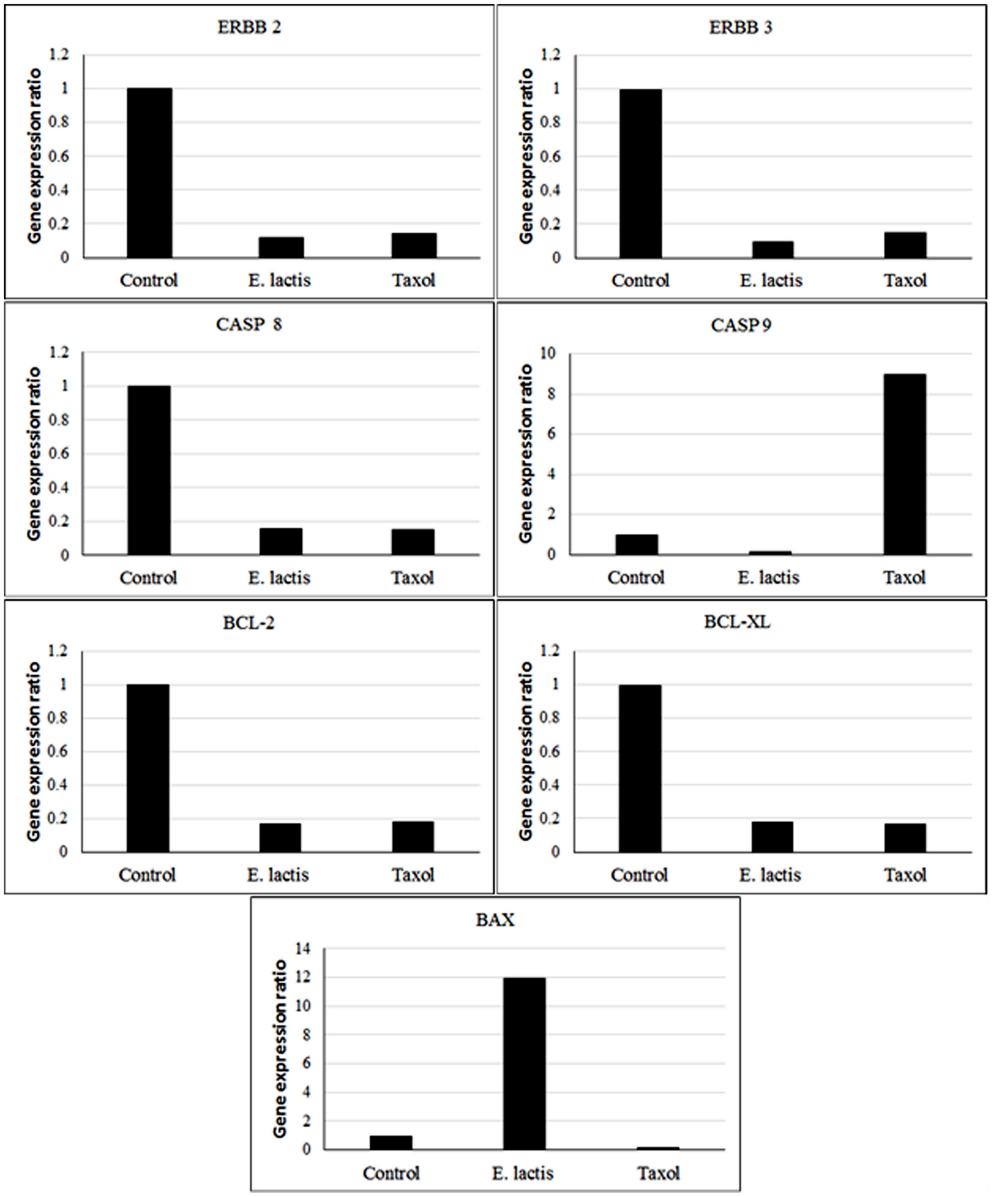
**Apoptosis related gene expression ratio in treated (50 μg/mL *E. lactis* secretion metabolites) and untreated control HeLa cells for 24 h**.

As shown in **Figure 4**, the expression of anti-apoptotic genes (ERBB 2 and ERBB3), intrinsic apoptosis blocker genes (BCL-2 and BCL-XL), and CASP 8 gene (starter gene in TNF-α apoptosis pathway) were significantly down-regulated by *E. lactis* IW5compared to untreated control group. The down-regulation in the mentioned genes by *E. lactis* IW5 was similar to Taxol^®^ but the expression of CASP 9 (starter gene in intrinsic apoptosis pathway) and BAX (crucial gene in extrinsic IL-3 mediated apoptosis pathway) genes was significantly different in *E. lactis* IW5 and Taxol treated groups (**Figure 4**). *E. lactis* IW5 up-regulated the expression of BAX gene whereas Taxol up-regulated the expression of CASP9 indicating different inducing pathways of apoptosis. *Lactobacillus paracasei* M5L can induce apoptosis in HT-29 cells through reactive oxygen species generation followed by CRT accompanied endoplasmic reticulum stress and S phase arrest (Hu et al., 2015). The molecular mechanisms of pro-apoptotic effects of human-derived *Lactobacillus reuteri* ATCC PTA 6475 has been previously investigated on myeloid leukemia-derived cells and findings have shown the down-regulation of nuclear factor-kappaB (NF-kappaB)-dependent gene products that mediate cell survival (Bcl-2 and Bcl-xL) related genes (Iyer et al., 2008). Findings of antitumor effects of cell-bound exopolysaccharides (cb-EPS) isolated from *Lactobacillus acidophilus* 606 on HT-29 colon cancer cells have shown the antitumourigenic effects through the induction of BAX gene (Kim et al., 2010). In addition, the human probiotic *Propionibacterium freudenreichii* could kill HT-29 colorectal adenocarcinoma cells through apoptosis *in vitro* via its metabolites (the short chain fatty acids, acetate and propionate; Lan et al., 2007). Furthermore, the investigation results of the effect of probiotic *Bacillus polyfermenticus* on the growth of human colon cancer cells including HT-29, DLD-1, and Caco-2 cells have illustrated that *B. polyfermenticus* can inhibit tumor growth and its anticancer activity occurs through suppressing ErbB2 and ErbB3 genes (Ma et al., 2010). Based on our findings, the induction of apoptosis by *E. lactis* IW5 is related to extrinsic IL-3 receptor pathway and it is deferent from Taxol’s apoptosis induction (intrinsic mitochondria apoptosis pathway).

## Conflict of Interest Statement

The authors declare that the research was conducted in the absence of any commercial or financial relationships that could be construed as a potential conflict of interest.
